# Curvature-enhanced Spin-orbit Coupling and Spinterface Effect in Fullerene-based Spin Valves

**DOI:** 10.1038/srep19461

**Published:** 2016-01-20

**Authors:** Shiheng Liang, Rugang Geng, Baishun Yang, Wenbo Zhao, Ram Chandra Subedi, Xiaoguang Li, Xiufeng Han, Tho Duc Nguyen

**Affiliations:** 1 Physics and Astronomy Department, University of Georgia, Athens, Georgia 30602, USA; 2 State Key Laboratory of Magnetism, Beijing National Laboratory for Condensed Matter Physics, Institute of Physics, Chinese Academy of Sciences, Beijing 100190, China; 3 Hefei National Laboratory for Physical Sciences at Microscale, Department of Physics, University of Science and Technology of China, Hefei 230026, and Collaborative Innovation Center of Advanced Microstructures, Nanjing University, Nanjing 210093, China

## Abstract

We investigated curvature-enhanced spin-orbit coupling (SOC) and spinterface effect in carbon-based organic spin valves (OSVs) using buckyball C_60_ and C_70_ molecules. Since the naturally abundant ^12^C has spinless nuclear, the materials have negligible hyperfine interaction (HFI) and the same intrinsic SOC, but different curvature SOC due to their distinct curvatures. We fitted the thickness dependence of magnetoresistance (MR) in OSVs at various temperatures using the modified Jullière equation. We found that the spin diffusion length in the C_70_ film is above 120 nm, clearly longer than that in C_60_ film at all temperatures. The effective SOC ratio of the C_70_ film to the C_60_ film was estimated to be about 0.8. This was confirmed by the magneto-electroluminescence (MEL) measurement in fullerene-based light emitting diodes (LED). Next, the effective spin polarization in C_70_-based OSVs is smaller than that in C_60_-based OSVs implying that they have different spinterface effect. First principle calculation study shows that the spin polarization of the dz^2^ orbital electrons of Co atoms contacted with C_60_ is larger causing better effective spin polarization at the interface.

Organic semiconductor (OSEC) has recently attracted great attention in the spintronics community since it possesses a long spin lifetime due to the reduced intrinsic spin-orbit coupling (SOC) caused by the light-weight elements, and relatively weak hyperfine interaction (HFI) associated with the symmetry of the π-electron orbital^1^,^2^. Therefore OSECs with high carrier mobility would show spin coherence that may be maintained over macroscopic distances. The motivation for searching materials with long spin diffusion length is to obtain large magnetoresistance (MR) in organic spin valves (OSVs)^3^,^4^,^5^, high performance of spin-polarized organic light emitting diodes^6^,^7^,^8^,^9^ and the realization of electrically-controlled spin-transport polarization devices such as spin-transistors^10^. An OSV consists of a thin layer of organic molecules or polymers sandwiched between two ferromagnetic (FM) contacts (see Fig. 1a); the resistance of the device depends on the relative magnetization configuration of the electrodes. The operation of the OSV would include spin injection and detection by magnetic electrodes, and spin transport accompanied by the spin relaxation in the organic interlayer; the former is related to the interfacial spin-polarization, dubbed spinterface^11^,^12^,^13^,^14^,^15^,^16^,^17^ which is influenced by the spin polarization of the FM electrode and the OSEC/electrode orbital hybridization, whereas the latter depends on spin relaxation time and charge diffusion coefficient in the organic spacer which defines the spin diffusion length of the material (see Fig. 1b).

So far, there exist several challenges on understanding spin loss mechanism and temperature dependence of spin diffusion length in OSECs^18^. Drew *et al.*^19^ using muon-spin spectroscopy found that the carrier spin diffusion length in an Alq_3_ molecule-based OSV is significantly quenched at a temperature above 100 K leading to unobservable MR at the higher temperature. In contrast, using the ferromagnetic resonance spin pumping technique Jiang *et al.* found the temperature independence of spin diffusion length in Alq_3_ film^20^. In addition, Kawasugi *et al.*^21^ recently found ~10% MR in a TPD-based OSV at all temperatures. Although the spin diffusion length was not measured, the result implies that spin diffusion length in TPD molecules is also insensitive with the device temperature. Also, it has been theoretically and experimentally demonstrated that the HFI plays a crucial role in all spin responses in PPV-based polymers^5^,^22^,^23^. However, Ando *et al.*^24^ recently showed that a pure spin current can be pumped from a ferromagnetic electrode into conducting polymers and can be detected using inverse spin Hall effect, where intrinsic SOC plays an important role. The study calls for the reconsideration of the role of SOC on the spin transport in OSECs even when the material does not contain heavy metal^24^,^25^. However, there is a limitation in their experimental technique: the presence of a large magnetic field during the measurement quenches the HFI leading to an extremely long spin diffusion length of greater than 200 nm at room temperature^26^. So far, there is very little effort to understand the effect of intrinsic SOC in conventional OSEC-based spin valves. Nevertheless, there are several studies of spin response in metal complex molecules where large intrinsic SOC from heavy metals is clearly dominant over other spin interaction mechanisms^27^. The effective way, we believe, to remove the strong effect of HFI thereby considering only SOC effect is to study fullerene-based spin valves^28^,^29^,^30^,^31^,^32^,^33^; the materials are composed of 99% naturally abundant ^12^C atoms that have spinless nuclei, and thus zero HFI. Since the intrinsic SOC in C_60_ is estimated about 10 mK, the fullerene is assumed to have a long spin relaxation time^34^. In addition, C_60_-based spin valves show relatively large magneto-resistance (MR) at room temperature^30^,^31^,^32^, which is promising for the organics-based spintronics applications. In contrast to conventional OSECs, C_60_ material shows its mechanical robustness against the metal penetration during the electrode fabrication^32^,^35^ and therefore is an ideal material to study *spinterface science*. However, various C_60_-based OSVs studies surprisingly show that the spin diffusion length in C_60_ varies from 10 nm to more than 100 nm, not significantly larger than in conventional OSECs^29^,^30^,^33^. It is still not clear whether there exist any other spin loss mechanisms other than intrinsic SOC in fullerene. Recently, the study of SOC strength in carbon-based materials such as graphene, carbon nanotube and fullerene has gained tremendous attention due to their non-trivial topological phase that induces a charge hopping between the orbitals in the *π* and *σ* bands between neighboring carbon atoms causing the so-called curvature SOC^34^,^36^,^37^,^38^. Perhaps, C_60_ and C_70_ fullerenes with quite distinctive topological phases (see Fig. 1c,d) may be the most sufficient choice for comparing the effect of curvature SOC on spin transport. *Such critical study in fact has not been empirically achieved yet in fullerene-based spin valves.* Nevertheless, Arbogast *et al.*^39^ firmly reported the stronger SOC in C_60_ compared to C_70_ molecules while studying their photophysical properties. In contrast, various electron paramagnetic resonance studies on the doped C_60_ and C_70_ either in solution or in solid forms show that their relative electron *g* value depends on the use of doping agents^40^,^41^.

In this paper, we systematically studied the spin diffusion length of C_60_ and C_70_ films and the spinterface effect by using MR response in fullerene-based spin valves. The buckyballs, C_70_ and C_60_, possess the same intrinsic SOC but might have different effective SOC strength caused by their different curved structures. We found that a spin diffusion length of C_70_ is above 120 nm, inevitably longer than that of C_60_ at all temperatures, presuming that the effective SOC in C_70_ is smaller. The difference in effective SOC strength between the fullerenes can be confirmed by the magneto-electroluminescence (MEL) study in fullerene-based light emitting diodes (LED) where the C_60_ MEL response shows wider width (implying stronger SOC strength) than C_70_ MEL response^3^,^42^,^43^. In addition, the effective spin polarization of the electrodes in the C60-based device is also larger. However, they share the same reduction trend with increasing temperature. The discrepancies can be explained by the structural difference between the molecules causing (i) different effective SOC strength, and (ii) the different electron orbital hybridization between the molecules and the ferromagnetic electrodes. The latter is verified by the density functional theory (DFT) calculation.

## Results & Discussion

**Figure 1:**
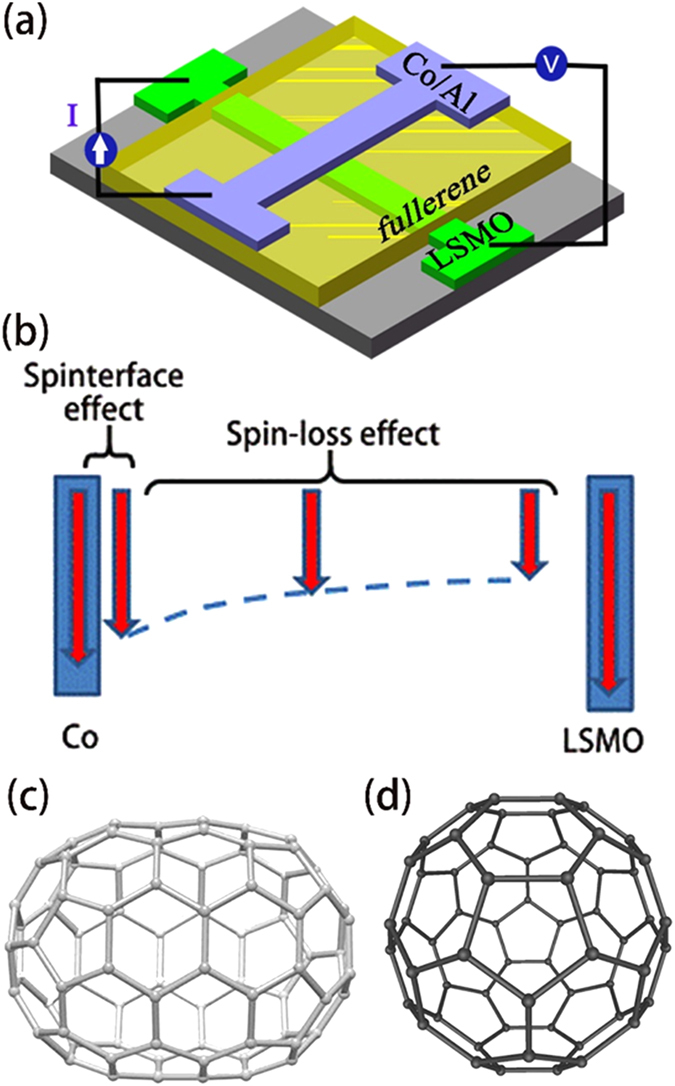
(**a**)Schematic representation of a typical device that consists of two FM electrodes and an OSEC spacer. An in-plane magnetic field, B, is swept to switch the magnetization directions of the two FM electrodes separately while the device resistance is measured using the four probe measurement technique. (**b**) The schematic representation of spinterface effect and spin-loss effect in organic spin valves. The arrows show the spin polarization of electrodes and transport electrons. Molecular structures of fullerene (**c**) C_70_ and (**d**) C_60_.

Figure 1a shows the OSV schematic representation of the devices used in this study where a 50 nm La_0.67_Sr_0.33_MnO_3_ (LSMO) film with ~100% spin polarization at low temperature is used as the bottom electrode and a 15 nm Co film with ~30% spin polarization is used as the top electrode^3^. It is possible to switch the relative magnetization of the ferromagnetic electrodes between parallel (P) and anti-parallel (AP) alignments, upon sweeping the external magnetic field, *B*. The device resistance *R(B)* at the field of *B*, is then dependent on the relative magnetization orientations. The MR response is commonly defined as: MR = [*R*(*B*) − *R*(*P*)]/*R*(*P*), where R(*P*) is the device resistance for the parallel magnetization configuration. Figure 1c,d show the chemical structures of C_60_ and C_70_. A C_60_ molecule has a spherical cage-like fused-ring structure with radius of 7.1 Å while a C_70_ molecule has a belt of 6 hexagons inserted in at the equator of a C_60_ molecule, resulting in an ellipsoid with short and long axes of 7.12 Å and 7.96 Å, respectively^44^,^45^. Therefore the C_60_ molecule has larger symmetry and curvature than the C_70_ molecule. We note that the fullerene films mostly show amorphous phase with an excellent surface roughness (see Fig. S1).

**Figure 2:**
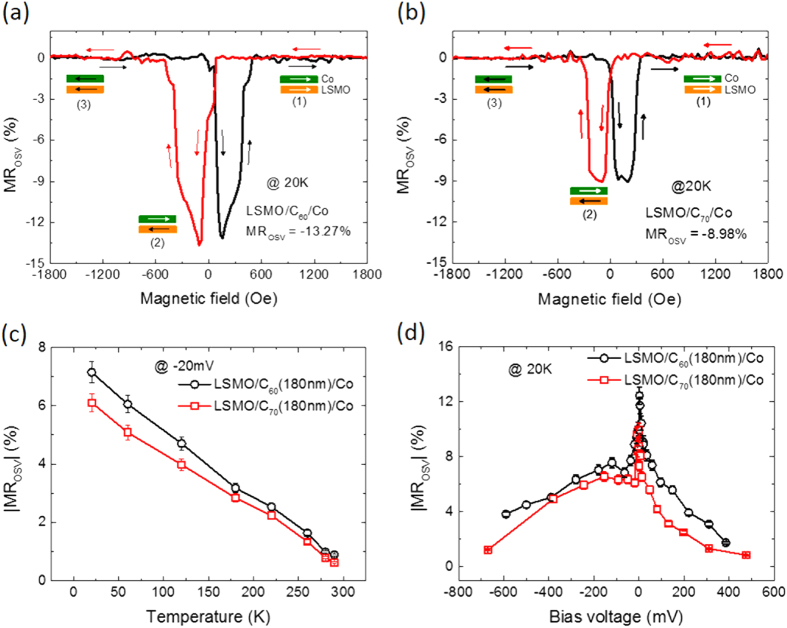
The magneto-resistance response of the spin-valve devices. MR loops of (**a**) LSMO(50 nm)/C_60_(120 nm)/Co(15 nm) and (**b**) LSMO(50 nm)/C_70_(120 nm)/Co(15 nm) OSV device measured at 20 K, with an applied bias voltage of −20 mV. The magnetization configurations are shown in the insets when the field is swept from positive field to negative field. (**c**) Temperature dependence of MR for the device of LSMO(50 nm)/C_60_(180 nm)/Co(15 nm) and LSMO(50 nm)/C_70_(180 nm)/Co(15 nm) OSV; (**d**) Bias voltage dependence of MR for the device of LSMO(50 nm)/C_60_(180 nm)/Co(15 nm) and LSMO(50 nm)/C_70_(180 nm)/Co(15 nm) OSV measured at 20 K. The bars in (**c**,**d**) show errors of the measurement.

Figure 2a,b show the MR loops of LSMO/C_60_(120nm)/Co/Al and LSMO/C_70_(120 nm)/Co/Al OSV devices measured at 20 K under an applied bias voltage of −20 mV, respectively. The background of all MR response (non-spin valve MR) caused by magnetic anisotropy of the electrodes is subtracted (see Fig. S2c,d for the original MR response)^46^,^47^. The IV characteristics at 20 K are shown in Fig. S2a. The red (black) curve denotes MR measurement while decreasing (increasing) magnetic field. The insets show the relative electrode magnetizations upon sweeping from positive *B*. The MR of the C_70_-based OSV is −9.0% while the MR of the C_60_-based OSV is −13.3%. The MR response in general follows the magnetic coercive field of the electrodes. Figure 2c shows the MR of LSMO/fullerene(180 nm)/Co OSVs device versus temperature. The MR decreases when temperature increases for both C_60_- and C_70_-based OSVs. This behavior is generic for all OSVs using the LSMO electrode^3^,^5^,^48^,^49^. However, the MR magnitude of C_60_ at this thickness is consistently larger than that of C_70_ at all temperatures at this fullerene thickness. The reduction of MR at higher temperature in general can be understood by the reduction of LSMO spin polarization and/or fullerene spin diffusion length.

Figure 2d shows the bias voltage dependence of MR. Firstly, the MR decreases with the increase in the junction voltage. There are two possible explanations for the bias-voltage dependence behavior for the reduction of MR at high bias voltages: (i) The Fermi energy which decides the density of state of injection spin polarization might be shifted under a relatively large applied bias voltage; therefore the effective spin polarization of LSMO (*P*_1_) and/or Co (*P*_2_) might be modified^48^; (ii) Since the two-step tunneling of electron from the ferromagnetic electrode into the fullerene interlayer favorably happened at large bias voltages does not conserve spin, the effective spin polarization of the electrode is smaller at larger bias voltage^5^,^50^. The asymmetry MR behavior versus applied voltage in our studies has been commonly observed by many groups^3^,^48^,^51^.

**Figure 3:**
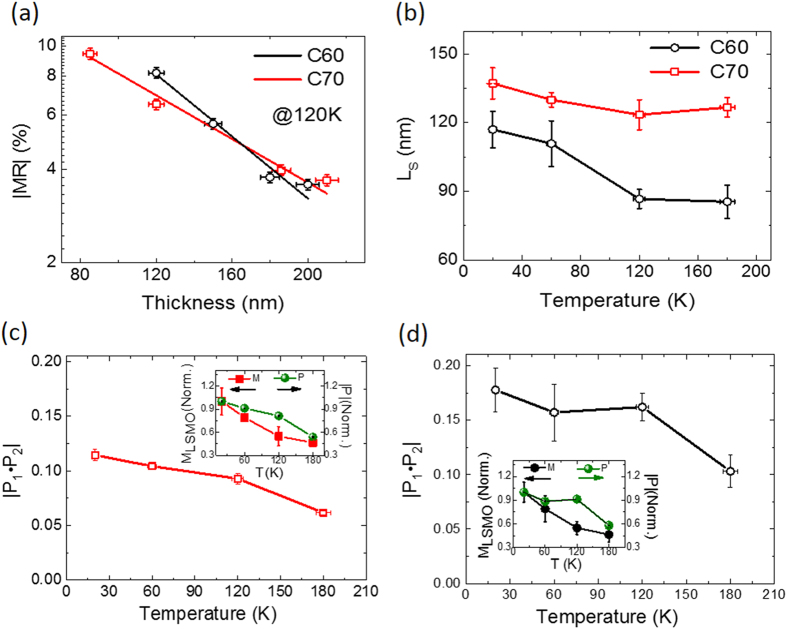
(a) Fullerence thickness dependence of MR at 120 K. The black and red open dots are measured MR of LSMO(50 nm)/C_60_/Co(15 nm) and LSMO(50 nm)/C_70_/Co(15 nm) OSVs, respectively. All MRs were taken at the junction voltage of −20 mV. The lines were fitted based on Eq. 1. (b) Temperature dependence of spin diffusion length, *L*_S_, of C_70_- and C_60_-based OSVs. The spin polarization, |*P*_1_·*P*_2_|, of (c) C_70_- and (d) C_60_-based OSVs. The insets show the normalized |*P*_1_·*P*_2_|, and LSMO magnetization versus temperature. The bars show errors of the measurement and fittings.

Figure 3a shows the thickness dependent MR of C_60_- (black dots) and C_70_- (red dots) based OSVs at 120 K presented in the log scale, where the MR in C_60_-based OSVs shows considerably stronger dependence than the MR in C_70_-based OSVs. We found that the MR magnitude in the C_70_-based OSVs is the largest at ~80 nm thickness (see Fig. S3a). Such behavior was previously reported in C_60_-based OSVs probably due to the morphology effect rather than the ill-defined organic layer caused by the cobalt inclusion^32^,^33^,^35^. The MR magnitude of fullerene-based OSVs gets smaller at the larger fullerene spacer thickness, d. We note that the ill-defined fullerene layer was found to be less than 15 nm (see Fig. S5). The smaller MR magnitude found in the larger spacer thickness can be explained by the reduction of the spin polarization of the injected carriers when travelling across the interlayer. Indeed, the MR magnitude in OSVs is normally described by the modified Jullière model^3^:


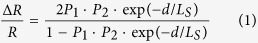


where *P*_1_ and *P*_2_ are the effective spin polarization at the interface of the magnetic electrodes; *L*_*S*_ is the spin diffusion length of the spacer.

In Fig. 3a, the black and red lines are the fitting data at 120 K using Eq. 1, where *P*_1_·*P*_2_ and *L*_*S*_ are the fitting parameters. For C_70_-based OSVs, |*P*_1_·*P*_2_| = (0.09 ± 0.01) and *L*_*S*_ = (123 nm ± 13 nm) while for C_60_-based OSVs, |*P*_1_·*P*_2_| = (0.16 ± 0.03) and *L*_*S*_ = (86 nm ± 8 nm). We fitted the thickness dependent MR magnitude at different temperatures using the same method (see Figs S3 and S4 for details). We found that the spin diffusion length *L*_*S*_ of C_70_ is indeed larger than that of C_60_ at all temperatures (Fig. 3b). In contrast, the effective spin polarization, |*P*_1_·*P*_2_|, of C_70_ is always smaller than that in C_60_ (see Fig. 3c,d). It should be noticed that *L*_*S*_ of fullerene weakly depends on temperature, in contrast to *L*_*S*_ in the Alq_3_ film reported by Drew *et al.*^19^, which decays quickly and is vanished at temperature higher than 100 K. We compare |*P*_1_·*P*_2_| with the magnetization near the surface of LSMO electrode measured by magneto-optical Kerr effect (MOKE)^3^. The insets of Fig. 3c,d show that the normalized effective spin polarization (P* = *|*P*_1_·*P*_2_|) and LSMO magnetization follow a similar temperature dependence except at 120 K. This indicates that the LSMO spin polarization might be a culprit for the MR reduction at high temperature. This is in agreement with the recent results reported by Kawasugi *et al.*^21^ who found that the MR was insensitive with the device temperature when the LSMO is replaced by the Co_2_MnSi heusler alloy. The obtained large spin diffusion length in fullerene-based OSVs at high temperature is similar to the value reported by Zhang *et al.*^30^ at room temperature. In the following sections, we explain the differences of spin diffusion length *L*_*S*_ and effective spin polarization |*P*_1_·*P*_2_| between C_70_ and C_60_ molecules.

In OSECs, since charge hopping is the main conduction mechanism, the spin diffusion length is generally described by the relation: *L*_*S*_* = *(*D·τ*_*S*_)^½^, where *D* is the carrier diffusion coefficient which is proportional to film mobility, and *τ*_*S*_ is the spin relaxation time^9^. Since the C_70_ has smaller mobility^52^ but larger measured spin diffusion length, its spin relaxation time must be longer than that in C_60_ leading to weaker SOC. Yu^23^ theoretically found that in conventional OSEC where HFI is dominant, *L*_*S*_ is linear with *D* and thereby depending on the material mobility. However, in the material such as fullerene where SOC is the dominant interaction, the *L*_*S*_ is given by^53^:


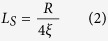


where *R* is the electron hopping distance, and 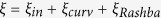 is the effective SOC strength where *ξ*_in_, *ξ*_curv_ and *ξ*_Rashba_ are the intrinsic, curvature and Rashba SOCs, respectively. The small electric field in the measurement leads to a neglegible *ξ*_Rashba_^34^. For the close-packed structures of C_70_ and C_60_, the nearest-neighbor distances between the adjacent molecules are ~10.1 Å and ~10.04 Å respectively^54^. This implies that the charge hopping distances are very similar in the materials. Equation 2 indicates that the spin diffusion length does not depend on the fullerene mobility. If the spin-related interaction contains only intrinsic SOC of carbon, the difference of spin diffusion length between C_60_ and C_70_ is simply the difference between the charge hoping distances. Figure 3b clearly shows that this is not the case and their effective SOC must contain the curvature-based SOC. From Eq. 2, the relative effective SOC strength between C_60_ and C_70_ molecules can be estimated as:


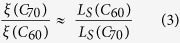


**Figure 4:**
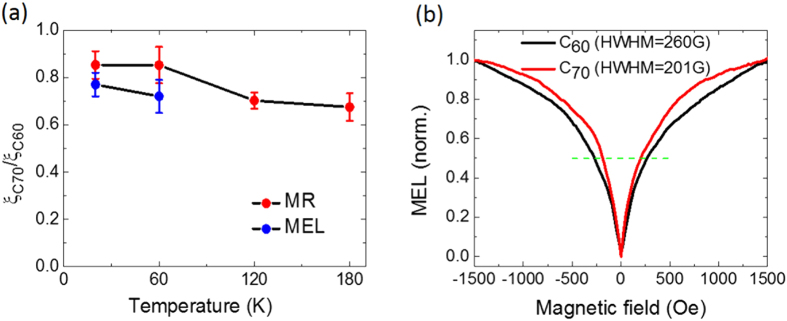
(a) Temperature dependence of relative SOC coupling strength between C_60_ and C_70_ molecules, ξ_C70_/ξ_C60_ taken from MR in OSVs and MEL in LEDs. The bars show errors from the calcullation. (**b**) Normallized magneto-electroluminescence (MEL) in fulerene LEDs at 20 K with the similar current density of about 1 mA/mm^2^. The line shows the half maximum of MEL.

From Eq. 3 and spin diffusion lengths in Fig. 3b, the relative SOC strength between C_60_ and C_70_ at various temperatures is calculated. Figure 4a shows that the effective SOC in C_70_ is smaller than that in C_60_ at all temperatures. Surprisingly, this ratio is slightly smaller at higher temperature. This might be related to their different orientational orders at different temperature^55^,^56^. Due to its higher symmetry, C_60_ might be easily rotated at higher temperature in all directions; this might prolong the effective electron hopping distance in C_60_ film, thereby reducing the spin diffusion length at high temperature. We note that our temperature dependence of L_S_ seems to conflict with Yu’s theory, in which the charge hopping distance, R strongly depends on the temperature and is convergent to the nearest neighbor hoping above a certain temperature (80 K for Alq_3_)^23^. This suggests that either the hopping distance is already saturated to the nearest neighbor hopping in our studied temperature range or the theory needs to be adjusted. Recently, based on the new experimental evidence of spin precession missing in OSECs^57^,^58^, Yu suggested a new spin tranport mechanism in organics utilizing the exchange-coupling between localized polarons, which can be much faster than polaron hopping. This spin-charge decoupling mechanism in principle can be used to explain the absence of the temperature denpendent spin diffusion length described in the Fig. 3b since incontrast to hopping transport, the exchange coupling is insensitive with the temperature. This sinario was supported by recent experiment by Jiang *et al.*^20^ where the spin precession of the pure spin current in Alq_3_ film was absent and the spin diffusion length was found to be independent on the temperature. However, the spin diffusion length in exchange-coupling mechanism strongly depends on the carrier density. This seems to be contradicted to the relatively slower decay of MR with large junction bias observed by many group^3^,^5^,^48^. Our result on SOC of C_60_ and C_70_ is consitent with result reported by Arbogast *et al.*^39^, who studied photophysical properties of the C_70_ compared to C_60_ molecules. They found that the intersystem crossing rate between singlet and triplet manifolds in C_60_ is larger than that in C_70_. This is a conclusive evidence that the SOC strength in C_70_ is weaker than in C_60_.

In order to further strengthen our conclusion in the SOC strength of the fullerene, we performed the MEL measurement on ITO/PEDOT/fullerene(180 nm)/Ca/Al LEDs. The MEL response has been used to evaluate the electron-spin related interactions for the last several decades^5^,^42^,^43^. The device structure and IV characteristics are shown in the Fig. S6. Figure 4b clearly shows the MEL response of the C_60_-based LED is broader than that of the C_70_-based LED at 20 K. Ehrenfreund *et al.* theorized the magnetic field effect on the polaron pair spin mixing dynamics and hence electroluminescence under the presence of the spin-orbit interaction^59^. The SOC Hamiltonian included in the over all Hamiltonian can be written as:


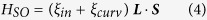


where ***L***, ***S*** are the orbital and spin angular momentum operators.

The calculation shows that the half width at half maximum (HWHM) of the magnetic response scales with the effective SOC strength, *ξ*_in_ + *ξ*_curv_. We estimate the effective SOC ratio of C_70_ over C_60_ is 0.77 which in agreement with the ratio shown in Fig. 4a. The MEL response at 60 K is shown in the Fig. S8 where much noisier MEL response were observed due to the weaker electroluminescence at higher temperature (Fig. S6). Neverthesles, the effective SOC ratio of about 0.7 is estimated. This is essentially in agreement with the study of SOC strengths by means of MR response in fullerene-based spin valves (Fig. 4a). At higher temperature, comparison is not conclusive due to the larger noise to signal ratio of the MEL response. We note that our result is in contrast to the recent theoretical calculation, in which the SOC caused by curvature in C_60_ is absent while the C_70_ has a large curvature SOC. The reason is that C_70_ molecule can be considered as a short nanotube capped by two semispheres that causes large curvature SOC in C_70_^34^. We note that the difference cannot come from the polycrystallites of films since the films are mostly amorphous (see Fig. S1b). In fact, the much larger SOC than calculated value in carbon nanotubes found by Steele *et al.*^36^ suggests that a considerable correction of theoretical calculations of curvature-based SOC should be done. The long spin diffusion length in fullerenes suggests that their effective SOC strength is weaker than the regular HFI strength in OSECs. This is in agreement with the long spin diffusion length obtained by Watanabe *et al.*^25^ where the material only has intrinsic SOC since the HFI is quenched^26^.

**Figure 5:**
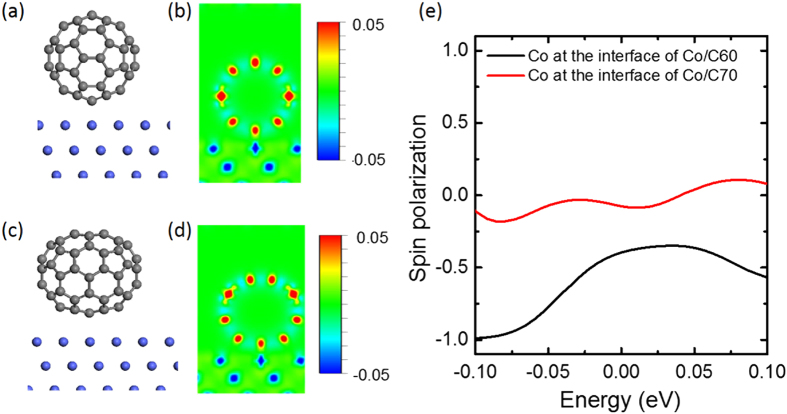
(**a**) Structure and (**b**) charge density difference isosurfaces of C_60_ on Co(111); (**c**) Structure and (**d**) charge density difference isosurfaces of C_70_ on Co (111). The isovalue is set to ± 0.05 Å^−3^. (**e**) The spin polarization of the *d*z^2^ orbital electrons of the cobalt contacted with C_70_ (red) and red C_60_ (black).

Next, we show that the spinterface effect between fullerene and Co might play the an important role in distinguishing the effective spin polarization between C_60_- and C_70_ based OSVs. To qualitatively understand the spinterface effect caused by the orbital hybridization at the interface, we performed the first-principle DFT calculations of the electron orbital absorption at the C_60_/Co and C_70_/Co interfaces where the ideal contact between the layer regardless of posible Co penetration was considered (see Figs S9 and 10, Suplementary Information for details). Figure 5a,b show the structure and charge density different isosurfaces of C_60_ on Co(111) respectively whereas Fig. 5c,d show the structure and charge density different isosurfaces of C_70_ on Co(111), respectively. The color bars describe the magnitude of charge density difference. The isovalue is set to ±0.05 Å^−3^. The sensitivity of the 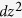 states comes from the fact that this orbital has a lobe of electronic density oriented perpendicular to the Co surface. States deriving from the 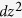 orbitals, which have long tails along the *z* direction, contribute significantly to the tunneling transport as compared to states deriving from other orbitals. Fig. 5e shows the spin polarization of the 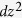 orbital electrons of the cobalt contacted with C_70_ (red) and C_60_ (black). The spin polarization of 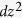 electrons of Co contacted with C_60_ is much larger than that of those contacted with C_70_ molecules. It means that the spinterface at the C_60_/Co interface is better. The calculation indeed explains our experimental results at different spinterfaces namely C_60_/Co and C_70_/Co. We note that the workfunction of LSMO surface studied by photoemssion spectroscopy was found to be sensitive to the organic solvents^60^, its spin polarization might potentially be affected by the solvents. However, various studies in spintronic devices showed that its interface and spinterface are very robust against mechanical and chemical reaction^2^,^3^,^61^. In addition, LSMO has been reported to be a good hole injector in LEDs made by polymers^6^,^7^ and small molecules^62^. Therefore, the different effective spin polarization of between the devices should come from Co/fullerene contact.

## Conclusion

We have successfully fabricated fullerene-based OSVs for studying spin injection into and spin transport through C_60_ and C_70_ amorphous films. We found that the spin diffusion length in C_70_ is considerably longer than that in C_60_. This indicates that its effective SOC and hence curvature SOC is smaller than that in C_60_. This was confirmed by MEL study of fullerene-based LEDs. In addition, the spin diffusion length in the materials is insensitive with temperature. Finally, the effective spin polarization in C_70_-based OSVs was found to be smaller than that in C_60_-based OSVs implying that they have different spinterface effect. This is confirmed by first principle calculation in which the spinterface caused by orbital electron hybridization at the cobalt/C_70_ interface is dominant causing smaller effective spin polarization at the interface.

## Methods

The OSVs were fabricated using C_60_ or C_70_ fullerenes as spacers sandwiched between LSMO (bottom magnetic electrode) and Co (top magnetic electrode). The device scheme is shown as Fig. 1a. LSMO films, having thickness of ∼50 nm and area of 5 × 5 mm^2^, were grown epitaxially on <100> oriented SrTiO_3_ substrates at 750 °C using magnetron sputtering technique, with Ar and O_2_ flux in the ratio of 1:1 in a pressure 4 Pa. The films were subsequently annealed at 800 °C for 2 hours in flowing O_2_ atmosphere before slowly cooled to room temperature, the average roughness of LSMO is about 1.0 nm (see Fig. S1(a)). The LSMO films were subsequently patterned using standard photolithography and chemical etching techniques. The LSMO films are already stable against oxidation; they can be cleaned and re-used multiple times without serious degradation. The fullerene spacer was thermally evaporated using an organic evaporation furnace with the evaporation rate of 0.2 Å /s at the base pressure of 2 × 10^−7^ torr; 15 nm cobalt (capped by 50 nm Al) top electrode was deposited onto the fullenere spacer using a shadow mask.The obtained active device area was typically about 0.2 × 0.4 mm^2^. The fabircation of fullerene light emitting diodes was started from paterning indium tin oxied (ITO) electrode, followed by spin-casting of hole transport layer, PEDOT:PSS. The deposition of fullerene and metals were performed in the similar procedure as in OSVs. All the fabrication was done in a nitrogen glove box where the oxygen and water levels are less than 0.1 ppm. Eventually, the OSVs and LEDs were mounted in the cold finger of a closed-cycle refrigerator whose temperature can be varied from 20 to 300 K. The MR was measured using the ‘four probe’ method in the presence of an in-plane magnetic field up to 3 kOe. The electroluminescence of LEDs was detected by a silicon photo diode while sweeping the magnetic field.

In the surface-MOKE method, the beam reflected from the sample passes a Glan-Thompson polarizing beam splitter, where it is separated into two orthogonal polarized beams which are focused by lenses onto diodes A and B of a diode bridge. The light intensities at the diodes, *I*_A_ and *I*_B_, and the difference signal *I*_A−B_ are simultaneously measured using a lock-in amplifier. A polarization balanced bridge detection technique cancels the influence of the background noise. The magnetization of the FM film is proportional to *I*_A−B_/(*I*_A_ + *I*_B_).

## Additional Information

**How to cite this article**: Liang, S. *et al.* Curvature-enhanced Spin-orbit Coupling and Spinterface Effect in Fullerene-based Spin Valves. *Sci. Rep.* **6**, 19461; doi: 10.1038/srep19461 (2016).

## Supplementary Material

Supplementary Information
