# Supplementary material for: Curvature-enhanced Spin-orbit Coupling and Spinterface Effect in Fullerene-based Spin Valves
Source: Sci Rep. 2016 Jan 20;6:19461. doi: 10.1038/srep19461 (PMC4726316; doi:10.1038/srep19461)
Supplement: Supplementary Information [file srep19461-s1.pdf]

**Supplementary Information**

# Curvature-enhanced Spin-orbit Coupling and Spinterface Effect in Fullerene-based Spin Valves

*Shiheng Liang[1], Rugang Geng[1], Baishun Yang[2], Wenbo Zhao[3], Ram Chandra Subedi[1], Xiaoguang Li[3], Xiufeng Han[2], Tho Duc Nguyen[1]**

*E-mail: ngtho@uga.edu

## (1) Morphology of LSMO and fullerene films.

The LSMO films having thickness of ~50 nm and area of 5×5 $mm^2$, were grown epitaxially on <100> oriented $SrTiO_3$ substrates at 750°C using magnetron sputtering technique, with Ar and $O_2$ flux in the ratio of 1:1 in a pressure 4 Pa. The films were subsequently annealed at 800 °C for 2 hours in flowing O2 atmosphere before slowly cooled to room temperature, the roughness of LSMO is about 0.2 nm, the morphology is shown in Fig. S1(a). The XRD spectra shown in Fig. S1(b) indicate the amorphous phase of the films.

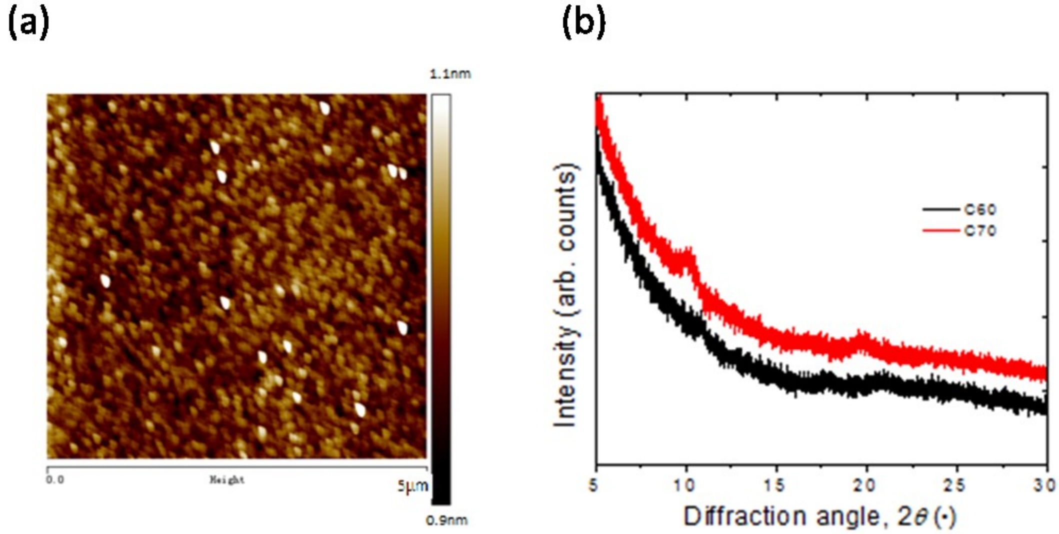


**Figure S1.** (a) Morphology of STO(001) substrate/LSMO(50nm) measured by atomic force microscopy. The average roughness in an area of 5µm×5µm is 0.22nm. (b) X-ray diffraction spectra of 180nm fullerene films.

**(2) IV characteristics of fullerene-based organic spin valves with 120 nm fullerene thickness and the magnetoresistance at -20mV bias voltage.**

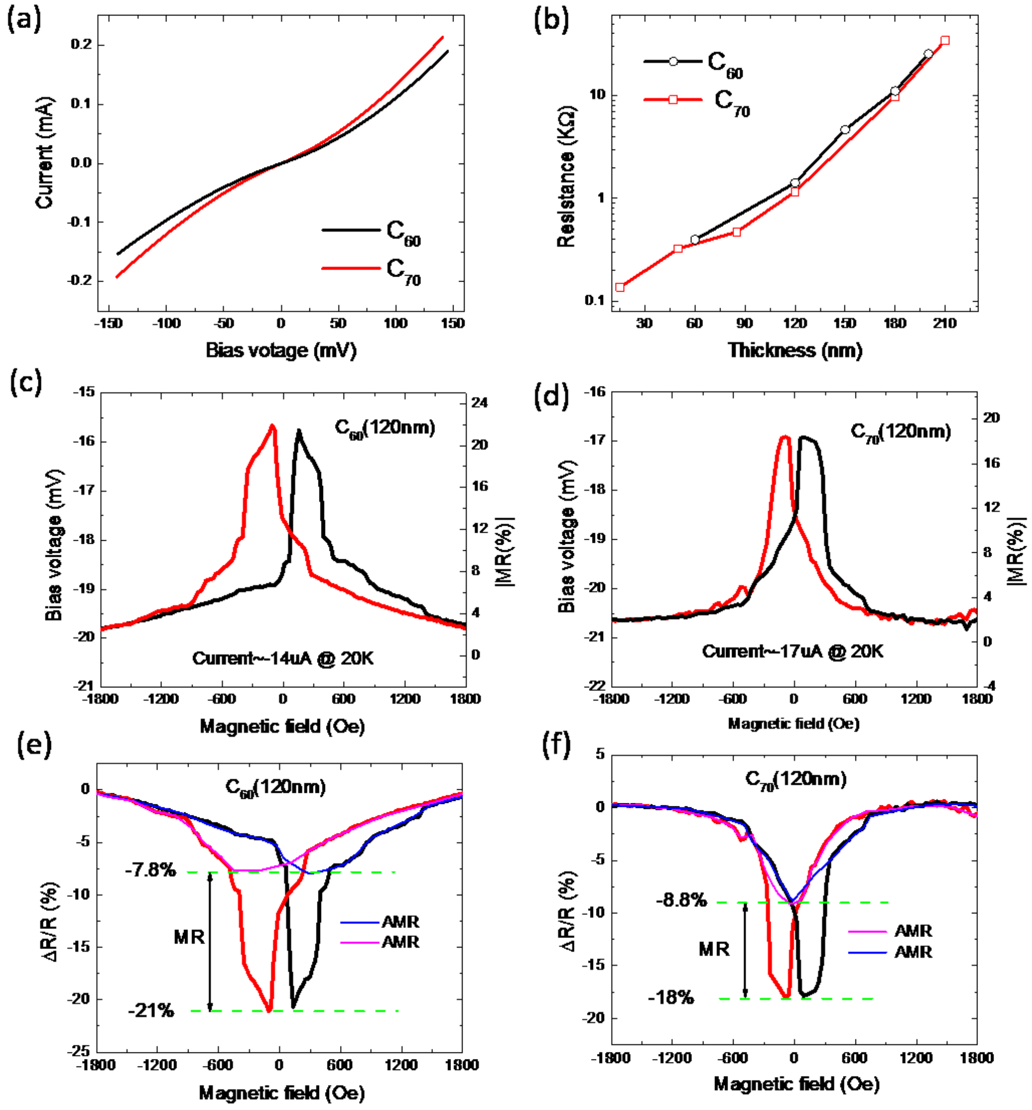


**Figure S2.** (a) The IV characteristics at the junction of LSMO/fullerene(120nm)/Co/Al. (b) Resistance of the devices measured with the junction voltage of -20 mV. The original measured bias voltage of (c) the C60-based OSV and (d) C70-based OSV with a certain applied current. The anisotropy magnetoresistance (AMR) was substracted from the total resistance change of (e) the C60-based OSV and (f) C70-based OSV. The insets show the subtraction background. All of the data were measured at 20K.

We note that the origin of the ”V shape” in **Fig. S2**(c) and (d) was discussed by Bobbert in terms of hyperfine interaction [1]. However, this interaction is absent in fullerene. Therefore, we can conclude that it is from the magnetic anisotropy of the LSMO as reported by Wu et al.[2]

**(3) Fullerene thickness dependent magnetoresistance**

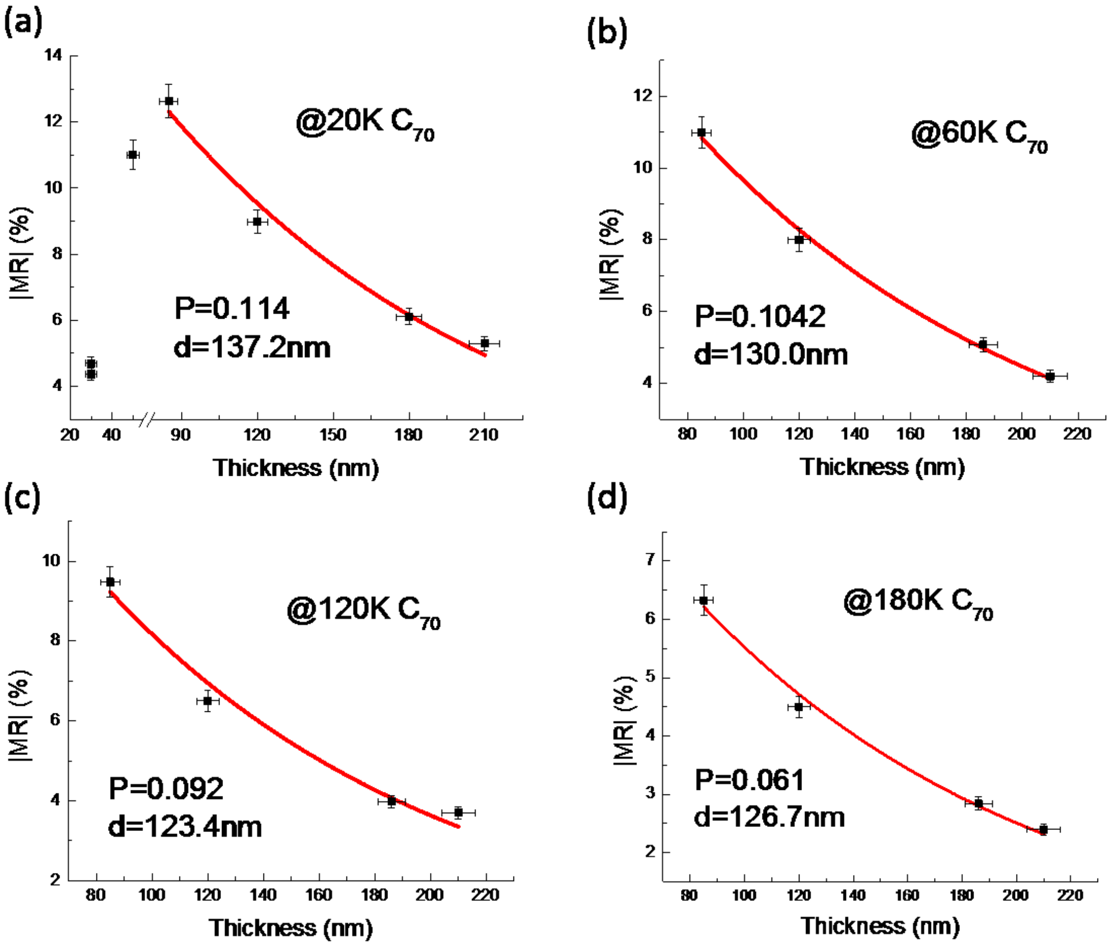


**Figure S3.** The thickness dependent MR of C70-based OSVs at (a) 20 K, (b) 60 K (c) 120 K and (d) 180 K. The error bars are shown. The lines show the fits using Eq. 1. Their fitting values are shown in the insets. We note that below 80 nm at 20 K the MR gets smaller.

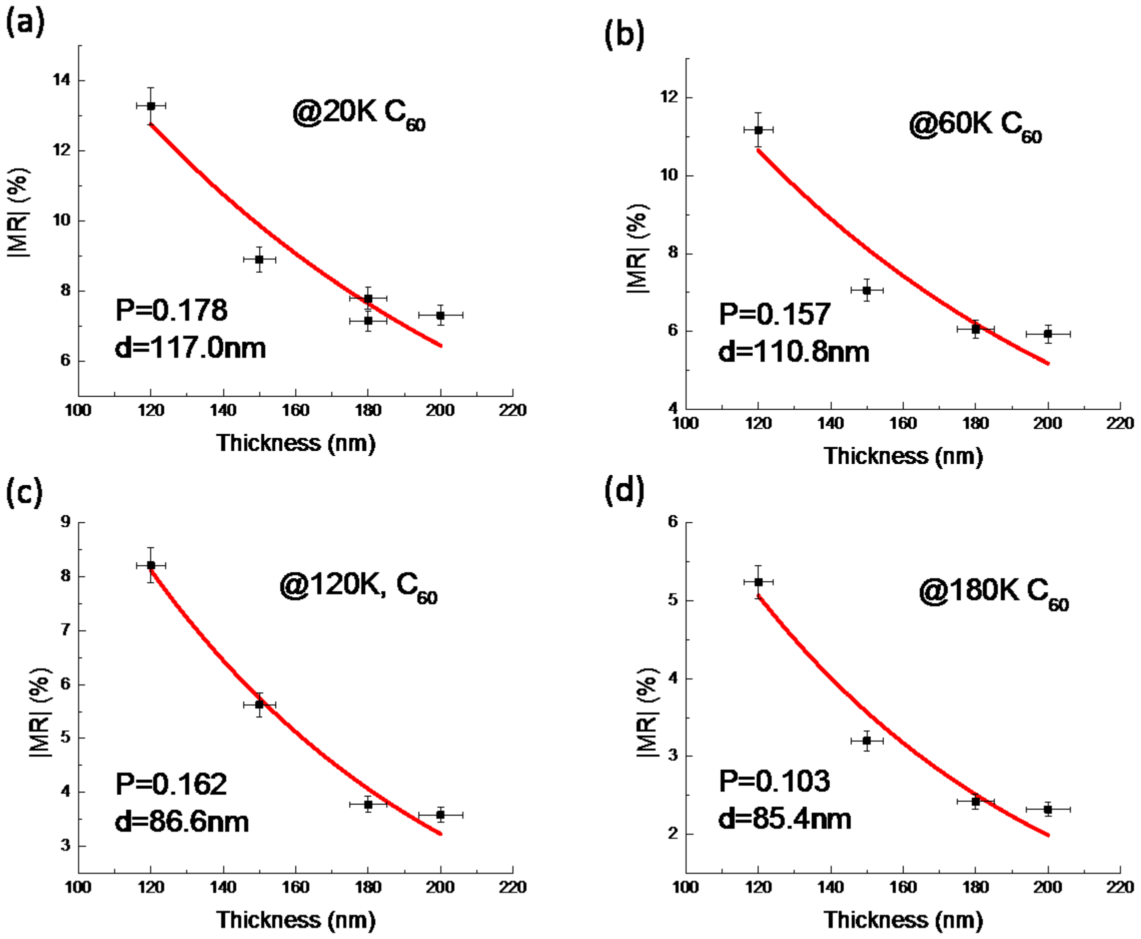


**Figure S4.** The thickness dependent MR of C60-based OSVs at (a) 20 K, (b) 60 K (c) 120 K and (d) 180 K. The error bars are shown. The lines show the fits using Eq. 1. Their fitting values are shown in the insets.

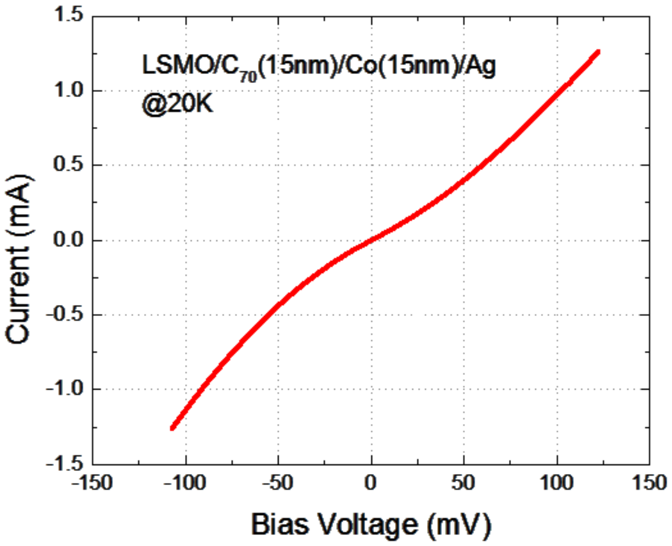


**Figure S5.** The IV characteristics of the junction of LSMO/fullerene(15nm)/Co/Al

Figure S5 shows the IV characteristics of an C70-based OSV with 15 nm thick spacer. The device does not show short circuit. This indicates the Co penetration depth would be smaller than 15 nm.

**(4) Magnetic field effect in ITO/PEDOT/fullerene(180nm)/Ca/Al LEDs**

The weak electroluminescence intensity of fullerene-based LEDs reduces significantly at high temperature (**Fig. S6**). The photoluminescence shown in **Fig. S6** indicates that the radiative singlet transistion is allowed in C60 and C70.

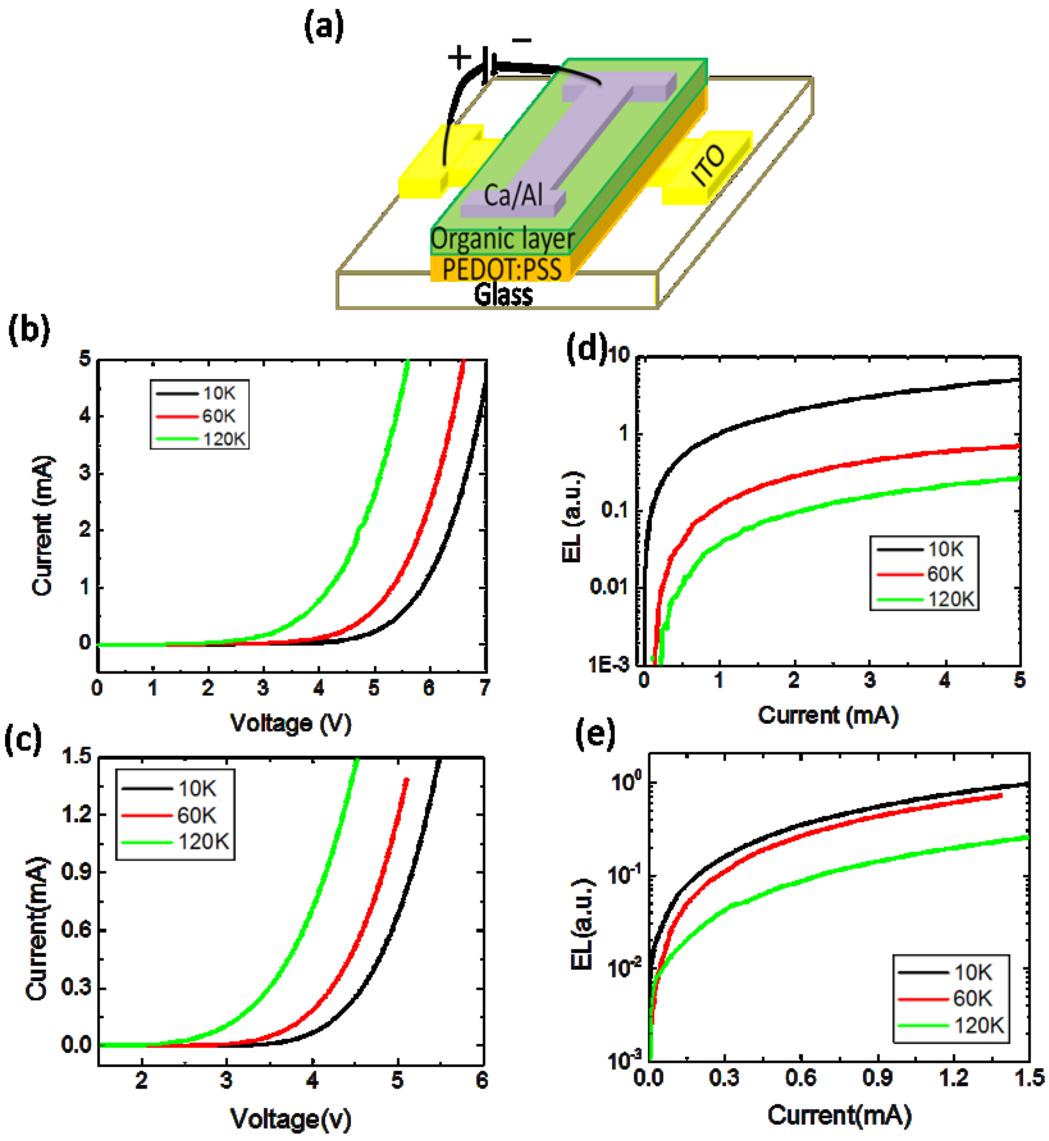


**Figure S6.** (a) The fulleren-based light emitting diode (LED) structure and meausurement. Current- voltage (IV) characteristics of (b) C70- and (c) C60-based LEDs with 180 nm thickness at various temperature. EL-I characteristics of (d) C70- and (e) C60-based LEDs.

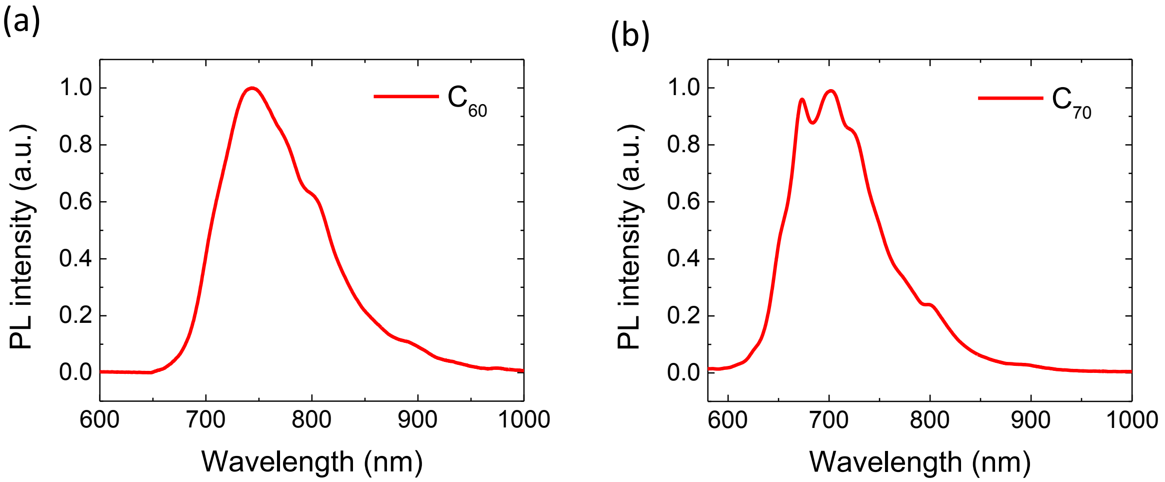


**Figure S7.** (a) Photoluminescence spectra of C60 and C70 films with excitation wave length of 405 nm at 20 K

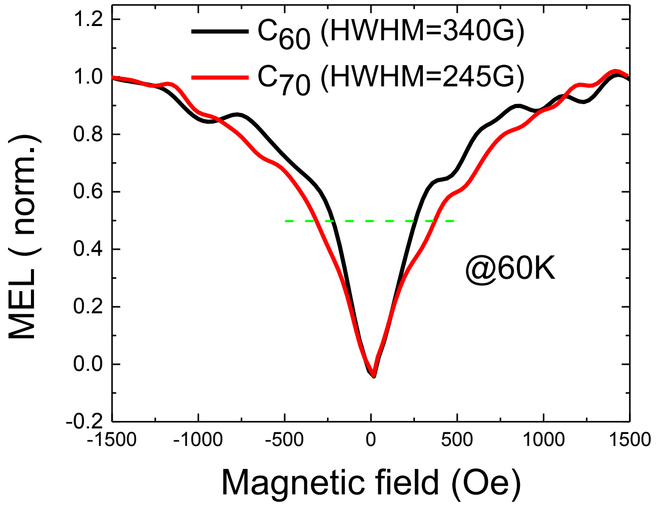


**Figure S8.** Magnetoelectroluminescence at 60 K. The data was smoothed using Origin software.

**(5) First-principles calculation of C60/Co and C70/Co**.

All calculations were performed within the density functional theory (DFT) calculations, as implemented in the Vienna Ab initio Simulation Package (VASP)[3,4]. The exchange and correlation are treated within the generalized gradient approximation (GGA) with Perdew-Burke-Ernzerhof (PBE)[5] functional and the ion-electron interaction was described by the projector-

augmented plane wave (PAW)[6] potentials. We use a plane wave kinetic energy cutoff of 500eV and a 3×3×3 Monkhorst-Pack k-point mesh was used for the Brillouin zone integrations. The atomic positions were optimized until the force on each atom was less than 0.01eV/Å.

In the electronic and magnetic properties of C60/Co(111) and C70/Co(111) interfaces calculations, a slab with seven layers of Co was selected. The atoms in the first and second layer of Co were relaxed, other atoms in Co substrate were fixed in all the simulations. Along the (001) direction, a 10Å vacuum was used to avoid the interaction between the adjacent layers.

When one C60 molecule adsorbs on this Co(111) surface, there are three high symmetry possible adsorbed sites considered: top, edge, and hollow. For the top site, the carbon atom in C60 is on top of Co atom; For the edge site, the Co atom is under the middle position of two carbons in C60; for the hollow site, the Co atom is under the center position of six-carbons rings of C60 molecule, as shwon in **Fig. S9** (a). When one C70 molecule adsorbs on this Co(111) surface, considering that when C70 molecular absorbing on the top site of Co, the long axis of C70 molecular will incline, so there are two high symmetry possible adsorbed sites considered: edge, and hollow, as shown in **Fig. S9**(b). By calculation of the binding energy $E_b$ between molecule and Co(111) surface, the stability of the adsorption structure was analyzed. $E_b$ is given by the following expression:

$$E_b = E_{mol} + E_{surf} - E_{mol+\mathrm{surf}} \tag{S1}$$

where $E_{mol}$ and $E_{surf}$ are the total energies of C60 or C70 molecule and the Co(111) surface, respectively. $E_{mol+\mathrm{surf}}$ is the energy of the molecule/Co(111) system. In our calculation of $E_b$ Eads, the molecule/Co(111) system, molecule, and the Co(111) surface were calculated by using the same calculation parameters. The edge site of both C60 and C70 are the most stable with distance of 1.867Å and 1.936Å, respectively, as shown in **Fig. S9**(a) and **Fig. S9**(b).

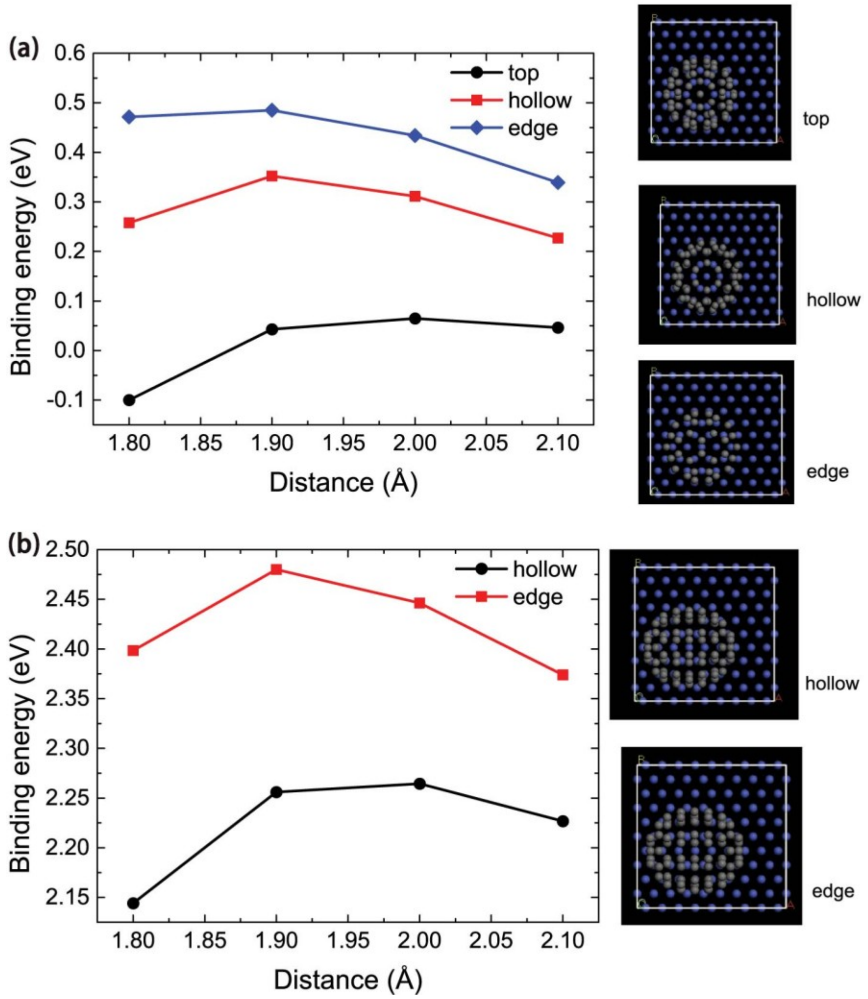


**Figure S9.** Typical high-symmetry adsorption configurations of (a) C60 and (b) C70 adsorbed on a Co(111) fcc terrace. The grey and blue balls represent carbon and cobalt, respectively.

**Figure S10** (a) shows the density of states of the Co connected with C60 molecules, and **Fig. S10** (b) shows the density of states of the Co connected with C70 molecules. Based on the density of states of the $dz^2$ orbital electrons, the spin polarization of the $dz^2$ orbital electrons were calculated, and shown in **Fig. 5**e in the main text.

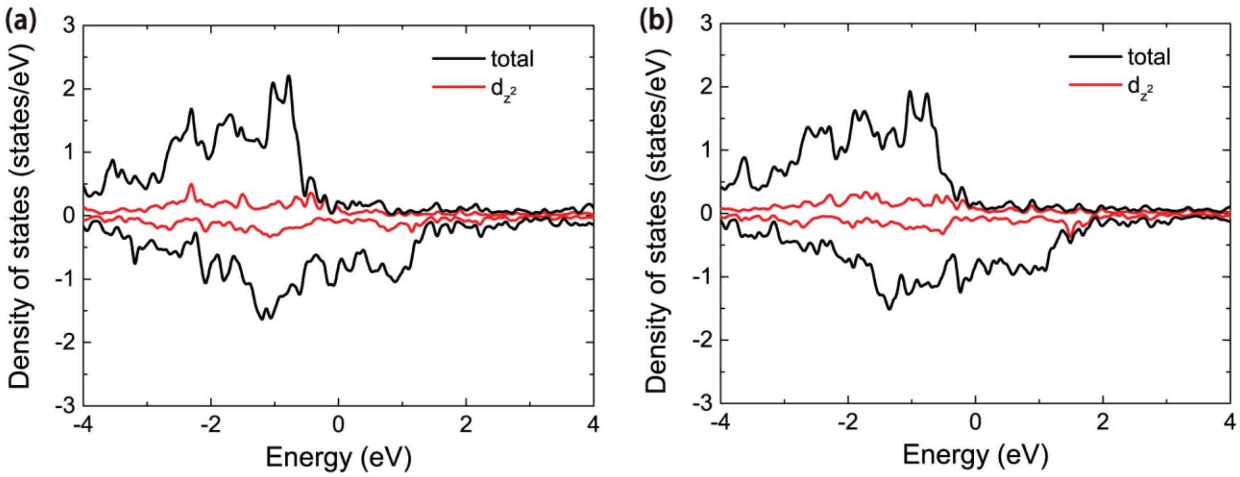


**Figure. S10.** (a)The density of states of the Co atom connected with C60 molecules, (b) the density of states of the Co atom connected with C70 molecules. The black curve indicate the density of states of total states and red curves indicate the density of states of the $dz^2$ orbital electrons.

References:

[1] Bobbert, P. A., Wagemans, W., van Oost, F. W. A., Koopmans, B. & Wohlgenannt, M. *Phys. Rev. Lett.* **102,** 156604 (2009).

[2] D. Wu, Z. H. Xiong, X. G. Li, Z. V. Vardeny, and Jing Shi Phys. Rev. Lett. **2005,** 95, 016802.

[3] G. Kresse and J. Hafner, *Phys. Rev. B* **1993**, *47*, 558; G. Kresse and J. Hafner, *Phys. Rev. B* **1994**, *49*, 14251.

[4] G. Kresse and J. Furthmüller, *Comp. Mater. Sci.* **1996**, *6*, 15; *Phys. Rev. B* **1996,** *54*, 11169.

[5] J. P. Perdew, K. Burke and M. Ernzerhof, *Phys. Rev. Lett.* **1996**, *77*, 3865.

[6] P. E. Blöchl, Phys. Rev. B: Condens. *Matter Mater. Phys.* **1994**, *50*, 17953.
